# Genome-wide identification and expression analyses of *CYP450* genes in sweet potato (*Ipomoea batatas* L.)

**DOI:** 10.1186/s12864-024-09965-x

**Published:** 2024-01-13

**Authors:** Xiongjian Lin, Binquan Tang, Zhenqin Li, Lei Shi, Hongbo Zhu

**Affiliations:** College of Coastal Agricultural Sciences, Guangdong Ocean University, Zhanjiang, 524088 China

**Keywords:** CYP450, Gene family, Sweet potato, Expression analysis

## Abstract

**Background:**

Cytochrome P450 monooxygenases (CYP450s) play a crucial role in various biochemical reactions involved in the synthesis of antioxidants, pigments, structural polymers, and defense-related compounds in plants. As sweet potato (*Ipomoea batatas* L.) holds significant economic importance, a comprehensive analysis of *CYP450* genes in this plant species can offer valuable insights into the evolutionary relationships and functional characteristics of these genes.

**Results:**

In this study, we successfully identified and categorized 95 *CYP450* genes from the sweet potato genome into 5 families and 31 subfamilies. The predicted subcellular localization results indicate that CYP450s are distributed in the cell membrane system. The promoter region of the *IbCYP450* genes contains various cis-acting elements related to plant hormones and stress responses. In addition, ten conserved motifs (Motif1-Motif10) have been identified in the *IbCYP450* family proteins, with 5 genes lacking introns and only one exon. We observed extensive duplication events within the *CYP450* gene family, which may account for its expansion. The gene duplication analysis results showed the presence of 15 pairs of genes with tandem repeats. Interaction network analysis reveals that *IbCYP450* families can interact with multiple target genes and there are protein-protein interactions within the family. Transcription factor interaction analysis suggests that *IbCYP450* families interact with multiple transcription factors. Furthermore, gene expression analysis revealed tissue-specific expression patterns of *CYP450* genes in sweet potatoes, as well as their response to abiotic stress and plant hormones. Notably, quantitative real-time polymerase chain reaction (qRT‒PCR) analysis indicated the involvement of *CYP450* genes in the defense response against nonbiological stresses in sweet potatoes.

**Conclusions:**

These findings provide a foundation for further investigations aiming to elucidate the biological functions of *CYP450* genes in sweet potatoes.

**Supplementary Information:**

The online version contains supplementary material available at 10.1186/s12864-024-09965-x.

## Introduction

Cytochrome P450 monooxygenases (CYP450s), named after the absorption band at 450 nm exhibited by their carbon-monoxide-bound form [1], are a ubiquitous class of enzymes found in various organisms, including bacteria, plants, and humans [2]. The discovery of the first *CYP450* gene occurred in rat liver microsomes [3, 4]. Subsequently, the X-ray crystal structure of CYP450 was obtained from bacterial CYP450cam [5]. As more *CYP450* genes were identified, the subfamily of *CYP450* genes expanded [6–8]. The *CYP450* family is present in a wide range of organisms, including plants, insects, animals, fungi, bacteria, and viruses [9]. Numerous *CYP450* genes, including approximately 16,000 in plants, have been identified across different organisms [10]. Gene structure analysis shows that members of this family all contain a conserved heme-binding domain with a sequence of FxxGxRxCxG [11]. Additional conserved domains are also present in CYP450 proteins. One such domain is the I-helix, which plays a role in both oxygen binding and catalysis on the distal side of the heme group. The sequence of the I-helix is characterized by the presence of A/G-X-E/D-T-T/S residues [12]. Furthermore, the PERF domain contains an arginine residue, while the K-helix consists of glutamate and arginine residues, forming the E-R-R triad. This triad is responsible for stabilizing the core structure of CYP450 enzymes [13]. Based on evolutionary relationships, plant CYP450s are divided into 11 families, with the smallest family number within each clan serving as its name [14]. The *CYP450* genes in plants can generally be divided into A-type and non-A-type. Among them, *CYP71* is A type, and other families are non-A type [15]. A considerable number of CYP450 proteins are involved in the biosynthesis and breakdown of diverse substances, encompassing plant hormones, secondary metabolites, and defense compounds [12]. Within this context, several *CYP450* families, including *CYP71*, *CYP85*, and *CYP72*, are responsible for conducting oxidation and rearrangement reactions that contribute to the biosynthesis of diterpenes. These diterpenes serve as essential components in the production of hormones, pharmaceuticals, aroma compounds, and food ingredients [16].

In *Arabidopsis*, the *CYP450* family ranks as the third-largest gene family and plays crucial roles in the synthesis of antioxidants, phytohormones, structural polymers, and defense-related compounds [17–25]. *AtCYP88A3* and *AtCYP88A4* play a role in gibberellin biosynthesis, and mutations in *CYP88* lead to a dwarf phenotype in barley and maize [12]. Recent studies have focused on *CYP450* genes involved in stress resistance and secondary metabolism, such as the gossypol biosynthesis pathway in cotton [26–29]. Additionally, *CYP450* genes have been implicated in drought tolerance, exemplified by *CYP86A2* in Arabidopsis and *CsCYP75B1* in citrus [30, 31]. Cold stress can also induce the expression of *CYP450* genes in perennial ryegrass, tall fescue, and roses [32, 33]. Furthermore, *CYP450* genes participate in the biosynthesis of jasmonic acid (JA), as demonstrated by *GmCYP82A3* in soybean and *DzCYP72As* in Dioscorea zingiberensis [34]. Multiple investigations have provided evidence supporting the involvement of *CYP716A* subfamily genes in regulating the biosynthetic pathway of triterpenoids. In the case of *Artemisia annua*, the genes *CYP716A14v2* and OSC2 (a multifunctional oxidosqualene cyclase) are responsible for the production of triterpenoids, which serve as constituents of the wax layer of the cuticle that covers the aboveground parts of the plants. Researchers suggest that specialized triterpenoids may serve a protective function against both biotic and abiotic stresses in *A. annua* [35]. Similarly, in sweet basil, *CYP716A52* and *CYP716A53* catalyze C-28 oxidation to yield oleanolic acid and unsolid acid. These compounds aid in the plant’s defense mechanism against stress [36]. These investigations propose that *CYP450* genes play a critical role in both plant growth and stress response. Previous investigations have successfully identified and carried out functional analysis on individual genes in the *CYP450* family of sweet potatoes. For example, *IbCYP73A1(IbC4H)* enhances the ability of plants to scavenge reactive oxygen species under stress. *IbCYP82D47* interacts with the carotenoid biosynthesis-related protein *IbGGPPS12*, increasing the content of carotenoids in transgenic sweet potatoes [37, 38]. However, a systematic identification and analysis of *CYP450* family members in sweet potatoes has not yet been conducted.

Sweet potato [*Ipomoea batatas* (L.)] is a vine plant belonging to the Convolvulaceae family. It is an important crop for food, feed, industrial raw materials, and new energy sources. Its edible enlarged storage root is a valuable source of nutrients and phytochemicals, making it widely cultivated [39]. These unique characteristics make it a staple food for humans, a feed source for animals, and a raw material source for the food and nonfood industries [40]. It is also used for the production of biofuels and alcohol [41]. With the development of sequencing technology, an increasing number of plant genomes and transcriptomes are being revealed, leading to a broader scope of research in this area. The sweet potato is one of the plants that has been extensively studied. In recent years, an increasing number of *CYP450* family genes have been discovered in crops, such as rice, soybean, and chili pepper [7, 42, 43]. However, there have been few reports on this gene family in sweet potatoes. In this study, the whole genome, evolutionary relationships, chromosomal localization, collinearity relationships, and expression patterns of the sweet potato *CYP450* gene family were identified and comprehensively analyzed using bioinformatics methods. The results provide a theoretical basis for understanding the functions of *CYP450* genes in sweet potatoes and for molecular breeding of sweet potatoes.

## Materials and methods

### Identification and physicochemical properties of *IbCYP* gene family members

The genomic data of sweet potatoes were obtained from the Ipomoea Genome Hub website (https://ipomoea-genome.org/) [44]. For the downloaded protein sequences, BLAST was used to construct a local database. In contrast, the gene and protein sequences of the Arabidopsis *CYP450* gene family were obtained from the Cytochrome P450 Homepage website (http://drnelson.uthsc.edu/CytochromeP450.html). A BLAST comparison of *CYP450* gene family protein sequences between sweet potato and Arabidopsis was conducted. At the same time, a hidden Markov model of the typical CYP450 family protein structure was downloaded from the Pfam database (http://pfam.sanger.ac.uk) [45], and HMMER software was used to search for the protein sequences [46] containing the characteristic domains (PF00067). The candidate proteins identified through the previously mentioned methods were further analyzed using Snapgene software. Incomplete reading frame sequences and redundant sequences were manually eliminated. The remaining candidate protein domains were validated using Pfam and the Conserved Domain Database (CDD) online analysis tools [47]. Gene sequences that did not contain the *CYP450* gene family domain or had incomplete CYP450 domains were removed from the analysis. Finally, 95 *IbCYP* genes were obtained and all the genes contained the FxxGxRxCxG characteristic domain. The coding sequence (CDS) and amino acid sequences of 95 *IbCYP* genes were corrected using the existing transcriptome sequencing results of sweet potato in our laboratory. The ExPASy ProtParam tool (http://web.ExPASy.org/protparam/) was used to predict protein physicochemical parameters [48]. Subcellular localization predictions were generated with BUSCA (http://busca.biocomp.unibo.it/) [49].

### Gene structure and conserved motif analysis

The exon–intron structure information of the candidate *IbCYP* was predicted by the online website GSDS2.0 (http://gsds.gao-lab.org/) [50]. The MEME online website (http://meme-suite.org/tools/meme) was used to predict the conserved domains in IbCYP450 protein sequences [51]. For this analysis, the number of motifs to be identified was set to 10, while default settings were adopted for other parameters and the results were visualized using TBtools. MEGA11 software [52] was used to perform multiple sequence alignment of 95 IbCYP proteins, and visualization was achieved with GeneDoc software.

### Phylogenetic analysis of IbCYP proteins

One representative member of each plant *CYP450* family was used for alignment and phylogenetic analysis. Members with identified functions in a family were preferentially selected for phylogenetic analysis. ClustalW was employed for multiple sequence alignment of CYP450 protein sequences between sweet potato, *Salvia miltiorrhiza*, pepper, tobacco, and *Arabidopsis*. The phylogenetic tree was constructed using MEGA11 neighbor-joining (NJ) with 1000 bootstrap replicates [52].

### Chromosomal localization, gene duplication, and synteny analysis

The locations of 95 *IbCYP* genes on chromosomes were obtained based on the information annotated for the sweet potato genome and analyzed through the Gene Location Visualization of TBtools [53]. *Arabidopsis*, tomato, pepper, maize, and rice downloaded from NCBI. Analysis of genome collinearity between sweet potatoes and these species was performed using MCScanX software [54]. Circos and Dual Synteny Plot in TBtools were used for visualized mapping of the collinear gene pairs [53].

### Cis-acting element analysis of *IbCYP* genes

The upstream promoter region (2,000 bp) of the *IbCYP* genes was extracted using TBtools software and submitted to the PlantCARE website (https://bioinformatics.psb.ugent.be/webtools/plantcare/html/) [55], which identified the cis-regulatory elements in the *IbCYP* genes. Then, the TBtools software was used to visualize the cis-regulatory element Fig. [53].

### Plant material and treatments

The sweet potato materials used in the experiment were obtained from the experimental field of the College of Coastal Agriculture, Guangdong Ocean University (21°15′N, 110°30′E).

After sweet potato seedlings were taken from the experimental field, the tuberous, pencil root, primary root, flower, fruit, and stem were covered with dry ice after being quickly frozen with liquid nitrogen. The tissues were then sent to Biomarker Technologies for total RNA extraction, library construction, and full-length transcriptome sequencing. Several strong branches grown consistently were selected and cultured with clean water for 10 days before being subjected to abiotic stress. During the development of adventitious roots in sweet potato shoots, the control group (CK) was maintained by continuing the culture with clean water. For salt stress treatment, the culture was continued with 200 mmol/L NaCl solution, and for drought stress treatment, 300 mmol/L mannitol solution was used. Each group was treated in triplicate with 3 branches per replicate. After 24 h of stress treatment, the primary roots, young stems, and leaves were taken and cooled by liquid nitrogen, covered with dry ice, and sent to Biomarker Technologies for transcriptomic sequencing.

### Protein-protein interaction (PPI) network construction

Using the default parameters, the online STRING database (https://string-db.org/) [56] was utilized to predict and execute potential protein-protein interaction networks using IbCYP proteins based on known Arabidopsis homologs. Cytoscape (V3.10.0) was used to visualize the resulting network [57].

### Transcriptome analysis

Five transcriptome bio project datasets were chosen for the *IbCYP450* gene expression profile analysis. Two bio project datasets (PRJNA511028 for hormone, and PRJNA987163 for cold) were downloaded from the NCBI database. Another three were our in-house (unpublished) sweet potato heat treatment, salt treatment, and drought treatment. Among them, “Xushu 18” was for hormonal treatment, clod-tolerant “Liaohanshu 21” and clod-sensitive “Shenshu 28” for cold treatment, heat-tolerant “Guangshu 87” and heat-sensitive “Ziluolan” for heat treatment and salt-tolerant and drought-tolerant “Guangshu 87” for salt and drought treatment. The *CYP450* expression was measured in fragments per kilobase of exon per million fragments mapped (FPKM). The heat maps of expression were constructed by TBtools software.

### Quantitative analysis of candidate *IbCYP* genes

The sweet potato (*I. batatas*) cultivar “Jishu 26” was used for qRT-PCR analysis in this study. Sweet potato plants were cultivated in a field at the experimental field of Guangdong Ocean University, Guangdong, China. For tissue expression, the flower, leaf, stem, primary root, firewood root, and tuberous root tissues were sampled from 3-month-old “Jishu 26” planted in the field. For the abiotic stress treatments, the twigs about 30 cm in length from 3-month-old filed-grown “Jishu 26” were cultured in the Hoagland solution for 14 days to treat: for salt stress treatment, the twigs were cultured in the Hoagland solution with 0 and 200 mM NaCl. For drought stress treatments, the twigs were cultured in Hoagland solution with 0 and 300 mM mannitol. The primary root, stem, and leaf samples were collected at 0, 8, 16, and 24 h after the treatments.

For qRT-PCR analysis, the 10 µL total reaction quantity of each sample contained 1 µL cDNA template, 0.5 µL (10µmol L-1) forward and reverse gene-specific primers, 5 µL 2×SYBR Green qPCR mix and 3 µL ddH2O. The qRT‒PCR reaction was conducted using the Bio-Rad system with the following thermal cycle conditions: 3 min of pre-degeneration at 95 °C, followed by 40 cycles of denaturation at 95 °C for 10 s and annealing at 60 °C for 30 s. The reaction was completed with a 5-second step at 65 °C and a cooling rate of 0.5 °C to reach 95 °C. Each sample was replicated 3 times, referring to Dingfa’s method [58] using the IbARF gene as an internal reference. We calculated relative transcript levels using the 2^−ΔΔCT^ method.

## Results

### Identification of cytochrome *CYP450* family genes in sweet potato

The 95 *IbCYP* genes are given new names according to the classification and naming principles of *CYP450*. The proteins produced by these genes have different lengths, with amino acids ranging from 381 (*IbCYP712A1*) to 873 (*IbCYP82D47*). Their weights also vary, from 42.39 kD (*IbCYP712A1*) to 98.35 kD (*IbCYP82D47*). The predicted isoelectric points of the proteins range from 5.52 (*IbCYP712A1*) to 9.45 (*IbCYP76G3*). Of these, 75 proteins have isoelectric points higher than 7, making them positively charged in acidic solutions. The proteins have different levels of hydrophilicity, ranging from − 0.384 (*IbCYP704A1*) to 0.075 (*IbCYP78A2*). Furthermore, according to BUSCA subcellular localization predictions, all IbCYP proteins are found in the endomembrane system (Table 1).

**Table 1:** Identification of *IbCYP* genes and analysis of physicochemical properties of proteins in sweet potato

Gene name	Accession Number	Chr	Size (aa)	Mv (kD)	pI	GRAVY	Predicted Location
IbCYP704A1	OR359876	LG14	534	61.35	7.88	-0.384	endomembrane system
IbCYP716D2	OR359873	LG9	471	53.89	9.32	-0.377	endomembrane system
IbCYP75B4	OR359797	LG12	634	71.89	6.17	-0.357	plasma membrane
IbCYP82D47	OR359867	LG11	873	98.35	8.8	-0.351	endomembrane system
IbCYP76G3	OR359864	LG9	563	64.38	9.45	-0.325	organelle membrane
IbCYP96A4	OR359841	LG1	509	59.05	9.12	-0.294	endomembrane system
IbCYP82G4	OR359849	LG2	483	55.42	8.59	-0.277	endomembrane system
IbCYP76G4	OR359865	LG9	439	50.33	9.21	-0.277	endomembrane system
IbCYP82G1	OR359848	LG6	523	60.30	8.55	-0.272	endomembrane system
IbCYP736A2	OR359869	LG9	469	54.11	8.77	-0.254	endomembrane system
IbCYP82G7	OR359850	LG3	468	53.82	7.15	-0.253	endomembrane system
IbCYP81Q3	OR359847	LG11	511	58.19	7.28	-0.253	endomembrane system
IbCYP72A1	OR359826	LG13	515	59.31	9.26	-0.247	endomembrane system
IbCYP736A4	OR359823	LG5	495	56.63	6.66	-0.245	endomembrane system
IbCYP73A1(IbC4H)	ADB65927.1	LG12	505	58.14	9.21	-0.233	endomembrane system
IbCYP81Q2	OR359846	LG11	512	58.19	8.82	-0.232	endomembrane system
IbCYP84A1	OR359851	LG7	516	58.52	6.38	-0.217	endomembrane system
IbCYP89A1	OR359852	LG7	522	59.43	8.9	-0.215	endomembrane system
IbCYP734A1	OR359832	LG11	513	58.66	7.7	-0.209	endomembrane system
IbCYP85A1	OR359858	LG7	465	53.16	9.13	-0.206	endomembrane system
IbCYP88A1	OR359834	LG14	493	56.84	8.53	-0.203	endomembrane system
IbCYP90C1	OR359835	LG5	510	57.59	9	-0.203	endomembrane system
IbCYP72A4	OR359828	LG13	520	59.71	8.4	-0.201	endomembrane system
IbCYP736A3	OR359822	LG5	503	57.47	8.39	-0.197	endomembrane system
IbCYP81Q1	OR359807	LG13	520	59.26	8.22	-0.186	endomembrane system
IbCYP72A7	OR359857	LG13	512	59.11	8.86	-0.185	endomembrane system
IbCYP701A1	OR359820	LG15	511	57.88	6.31	-0.184	endomembrane system
IbCYP97B1	OR359843	LG8	584	65.30	7.53	-0.184	endomembrane system
IbCYP71D8	OR359794	LG13	498	56.74	6.42	-0.183	endomembrane system
IbCYP716D3	OR359874	LG1	455	51.9	9.13	-0.177	endomembrane system
IbCYP96A3	OR359840	LG1	519	60.15	6.68	-0.175	endomembrane system
IbCYP87A1	OR359833	LG8	479	54.88	8.96	-0.174	endomembrane system
IbCYP76C2	OR359799	LG1	499	56.15	9.11	-0.171	endomembrane system
IbCYP81Q4	OR359808	LG10	511	58.41	8.56	-0.17	endomembrane system
IbCYP82F1	OR359810	LG4	514	58.61	6.64	-0.167	endomembrane system
IbCYP82G5	OR359814	LG2	541	60.85	6.02	-0.162	endomembrane system
IbCYP97A1	OR359842	LG8	625	69.92	6.23	-0.161	chloroplast outer membrane
IbCYP81H1	OR359805	LG7	454	51.84	8.85	-0.161	endomembrane system
IbCYP72A3	OR359827	LG8	524	59.75	8.9	-0.16	endomembrane system
IbCYP82G6	OR359815	LG3	519	58.68	8.46	-0.159	endomembrane system
IbCYP71D4	OR359790	LG6	509	57.53	8.77	-0.159	endomembrane system
IbCYP716A1	OR359860	LG7	481	54.22	9.17	-0.156	endomembrane system
IbCYP736A1	OR359855	LG14	496	56.78	8.06	-0.148	endomembrane system
IbCYP76G1	OR359844	LG6	506	57.68	8.14	-0.148	endomembrane system
IbCYP71D5	OR359791	LG6	505	56.42	8.28	-0.145	endomembrane system
IbCYP716D1	OR359836	LG2	483	55.16	9.09	-0.145	endomembrane system
IbCYP75B2	OR359795	LG8	514	58.25	8.79	-0.142	endomembrane system
IbCYP736A6	OR359825	LG15	502	56.53	9.13	-0.142	endomembrane system
IbCYP72A2	OR359856	LG13	516	58.91	7.21	-0.14	endomembrane system
IbCYP83A1	OR359817	LG15	432	49.44	6.78	-0.137	organelle membrane
IbCYP714A1	OR359831	LG5	537	60.82	9.45	-0.134	endomembrane system
IbCYP82G3	OR359813	LG2	521	59.36	8.31	-0.132	endomembrane system
IbCYP735A1	OR359871	LG5	503	57.58	8.46	-0.129	endomembrane system
IbCYP706A1	OR359853	LG4	511	57.96	6.52	-0.128	endomembrane system
IbCYP712A1	OR359821	LG6	381	42.39	5.52	-0.124	endomembrane system
IbCYP86A1	OR359861	LG2	543	61.14	6.77	-0.124	endomembrane system
IbCYP72A6	OR359830	LG13	514	58.89	8.27	-0.123	endomembrane system
IbCYP82F2	OR359811	LG4	522	59.37	8.22	-0.122	endomembrane system
IbCYP89A2	OR359819	LG7	516	58.66	8.4	-0.121	endomembrane system
IbCYP82C1	OR359809	LG7	529	59.99	7.99	-0.119	endomembrane system
IbCYP79A1	OR359845	LG6	531	60.16	8.34	-0.116	endomembrane system
IbCYP75B3	OR359796	LG8	518	58.98	9.21	-0.116	endomembrane system
IbCYP81K1	OR359806	LG15	505	57.80	5.85	-0.108	endomembrane system
IbCYP82G8	OR359816	LG2	536	60.92	6.9	-0.105	endomembrane system
IbCYP714E1(GAs)	OQ184947.1	LG3	518	58.04	7.18	-0.104	endomembrane system
IbCYP716A2	OR359872	LG1	445	50.23	8.72	-0.103	endomembrane system
IbCYP736A5	OR359824	LG1	518	58.41	7.68	-0.101	endomembrane system
IbCYP96A2	OR359839	LG1	534	60.43	8.26	-0.099	endomembrane system
IbCYP76G2	OR359800	LG6	506	57.01	8.41	-0.096	endomembrane system
IbCYP706A2	OR359854	LG10	513	57.63	7.19	-0.093	endomembrane system
IbCYP72A8	OR359870	LG11	516	58.90	8.73	-0.09	endomembrane system
IbCYP76C1	OR359798	LG1	533	60.39	8.73	-0.087	endomembrane system
IbCYP77A1	OR359801	LG7	518	57.78	8.84	-0.082	endomembrane system
IbCYP78A1	OR359866	LG1	484	54.66	8.65	-0.079	endomembrane system
IbCYP82G2	OR359812	LG5	537	60.53	7.7	-0.078	endomembrane system
IbCYP72A5	OR359829	LG13	519	59.64	9.26	-0.068	endomembrane system
IbCYP75B1(IbF3’H)	MT557577.1	LG14	453	50.27	8.98	-0.066	endomembrane system
IbCYP707A1	OR359859	LG15	474	53.71	9.27	-0.062	endomembrane system
IbCYP83A2	OR359818	LG15	503	57.80	8.01	-0.058	endomembrane system
IbCYP71D3	OR359789	LG6	494	55.52	6	-0.054	endomembrane system
IbCYP71D7	OR359793	LG3	435	49.05	5.66	-0.05	endomembrane system
IbCYP94A2	OR359863	LG3	489	56.3	8.99	-0.049	endomembrane system
IbCYP71A1	OR359786	LG8	458	52.28	6.05	-0.048	endomembrane system
IbCYP86A2	OR359862	LG5	542	61.45	8.81	-0.041	endomembrane system
IbCYP77B1	OR359802	LG14	508	57.26	9.08	-0.041	endomembrane system
IbCYP714E2(GAs)	OQ184948.1	LG12	521	57.88	8.77	-0.039	endomembrane system
IbCYP96A1	OR359838	LG13	516	58.93	7.61	-0.037	endomembrane system
IbCYP71D1	OR359787	LG6	512	57.56	7.01	-0.031	endomembrane system
IbCYP94C1	OR359875	LG11	507	57.65	8.28	-0.03	endomembrane system
IbCYP71D6	OR359792	LG4	503	57.09	8.82	-0.029	endomembrane system
IbCYP71D2	OR359788	LG2	514	57.72	6.72	-0.028	endomembrane system
IbCYP94A1	OR359837	LG3	541	61.01	8.3	-0.027	endomembrane system
IbCYP82G9	OR359868	LG3	505	56.55	6.98	-0.007	endomembrane system
IbCYP78A3	OR359804	LG5	567	63.88	8.92	0.052	endomembrane system
IbCYP78A2	OR359803	LG8	536	59.43	9.18	0.075	endomembrane system

*MW *Molecular weight, *pI *isoelectric point, *GRAVY *Grand average of hydropathicity score

### Motif compositions and gene structure of the *IbCYP* genes

**Fig. 1:**
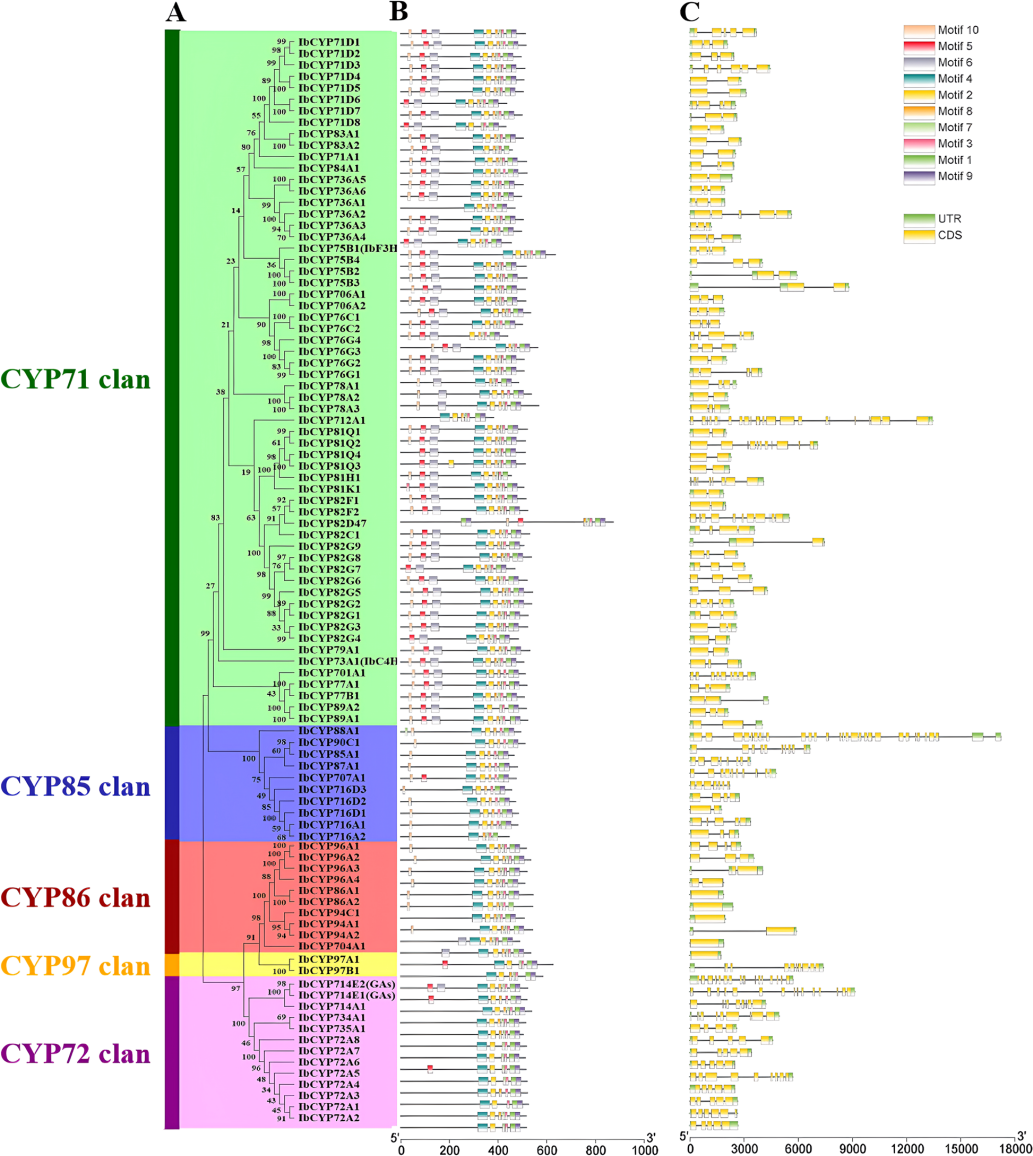
Phylogenetic tree, conserved motif, and gene structure of the *IbCYP* family in sweet potato. **A** A neighbor-joining (NJ) phylogenetic tree of sweet potato protein with 1000 bootstrap replicates was constructed based on the full-length sequence in MEGA11. **B** Distribution of conservative motifs in IbCYP proteins with colored boxes representing motifs 1-10 and scale representing 50 amino acids. **C** The genetic structure of the *IbCYP* gene, including introns (black line), exons (yellow rectangle), and untranslated regions (UTRs, green rectangle), with the scale representing 1 kb

According to the classification principles of the *CYP450* family, we have divided the identified 95 IbCYP proteins into 5 families (Fig. 1A). After analyzing the CYP450 protein sequence of sweet potato using the MEME online tool, ten conserved motifs were predicted (Fig. 1B). Different IbCYP proteins showed variations in the number and distribution of these motifs. Each gene had between six and ten motifs, and all IbCYP proteins had a conserved heme-binding domain Motif 1. The C-terminal region of the IbCYP protein was highly conserved, with Motif 2 and Motif 3 commonly found in most proteins, while the N-terminal region was less conserved. The majority (86.7%) of A-type CYP450 proteins contained all ten motifs, while non-A-type CYP450 proteins typically had between six and nine motifs. Motif 10 was not found in the *CYP97* or *CYP72* families, and motif 5 was absent in the CYP86 family. This suggests that the *IbCYP* gene family shows both a high level of conservation and some differences. Different subfamilies have distinct types of motifs, which may be related to the various biological functions of genes within each subfamily.

We studied the structure of the coding sequences of all 95 *IbCYP* genes and found that the *CYP450* gene family members of sweet potato had 1 to 15 coding sequences. The number of introns varied from 1 to 3, indicating that there was significant variation in the gene structure of *IbCYP* genes (Fig. 1C), the yellow box represents the coding sequence (CDS) of the *CYP450* gene family members.

### Conserved sequence alignment of the *IbCYPs*

**Fig. 2:**
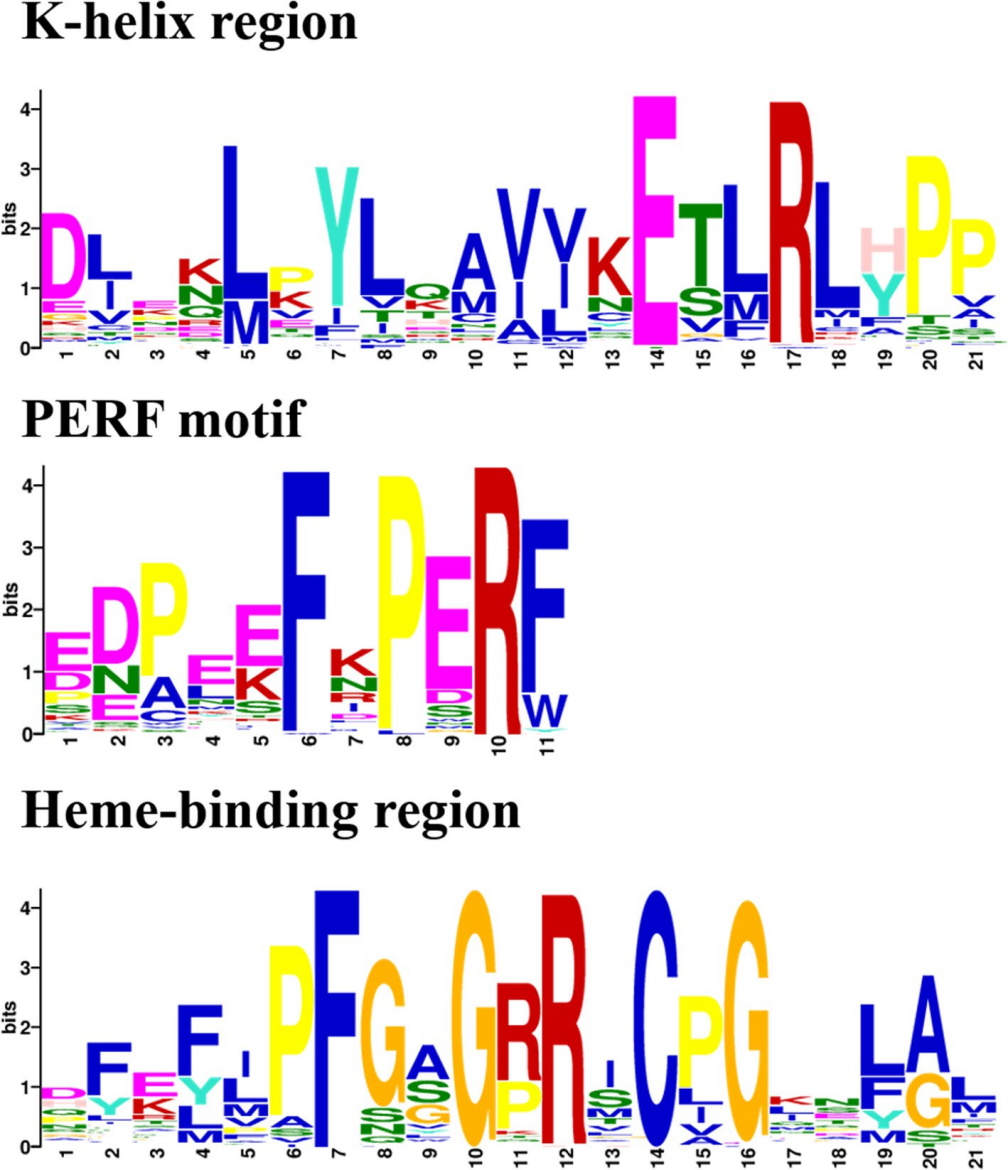
Conserved domains of sweet potato IbCYP proteins. Alignment of conservative motifs generated by the MEME online website for the 3 protein domains

 Multiple sequence alignment was performed for 95 IbCYP proteins using ClustalW. The results showed that all the IbCYP proteins have a highly conserved heme-binding region at the C-terminal end. The vast majority of IbCYP proteins (95.7%) exhibit the presence of all three conserved domains (Fig. 2), namely the K-helix region (ExxR), the PERF motif (PERF), and the heme-binding region (FxxGxRxCxG).

### Phylogenetic analysis of IbCYP proteins

**Fig. 3:**
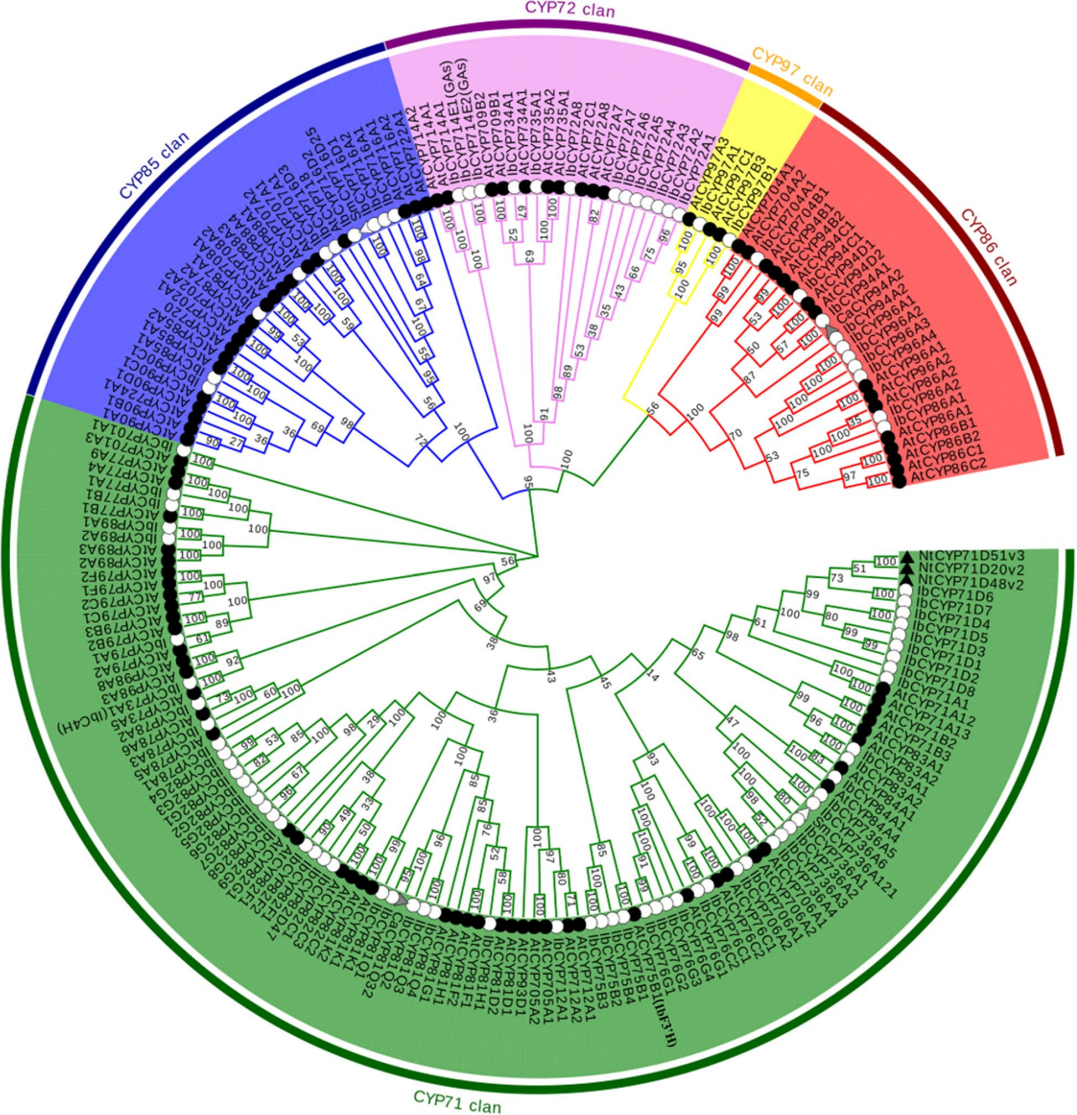
Phylogenetic Trees of CYP450 Proteins for Sweet Potato, Capsicum, Salvia miltiorrhiza, tobacco, and Arabidopsis. Arabidopsis CYP450 protein sequences were downloaded from the Cytochrome P450 Homepage website. A phylogenetic tree was constructed by the neighbor-joining method based on MEGA11 with 1000 bootstrap replicates. The tree was divided into 5 families represented by outer rings with different colors, black circles, white circles, black triangles, gray triangles, and white triangles representing the Arabidopsis, sweet potato, tobacco, capsicum, and Salvia miltiorrhiza CYP450 proteins

We constructed a phylogenetic tree containing 201 *CYP450* genes from five species (*I. batatas*, *A. thaliana*, *C. annuum*, *N. tabacum*, and *S. miltiorrhiza*) using MEGA software. We divided all of the *CYP450* genes into one of two major clades: A type, which contained the *CYP71* family, and non-A type, which contained 4 clans (Fig. 3).

### Chromosomal locations and gene duplication analysis

Gene duplication is recognized as a prominent driver in the evolutionary process of genomes and genetic systems [59]. Two main types of gene duplication, namely tandem and segmental duplication, can give rise to numerous gene families [60]. Tandem duplication occurs when multiple members of a gene family are found within the same intergenic region or neighboring intergenic regions [61, 62]. On the other hand, segmental duplication involves the duplication of multiple genes through polyploidy events, often followed by chromosomal rearrangements [63].

**Fig. 4:**
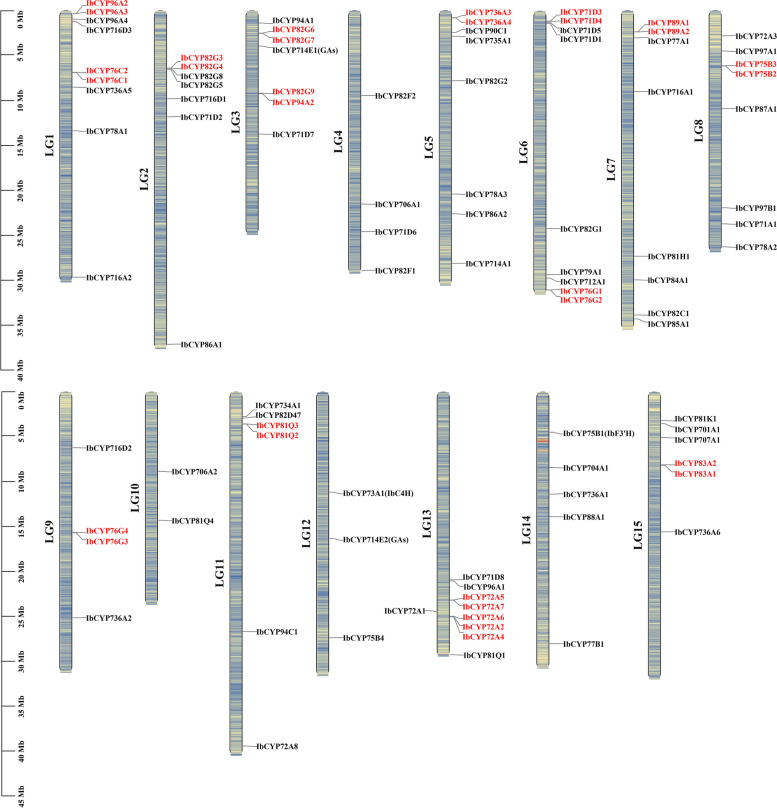
Locations of *IbCYP* genes on chromosomes. The basic unit indicated a chromosome length of 5.0 Mb. For each chromosome, the number is labeled on the upper side with red indicating a gene pair with tandem duplication

**Fig. 5:**
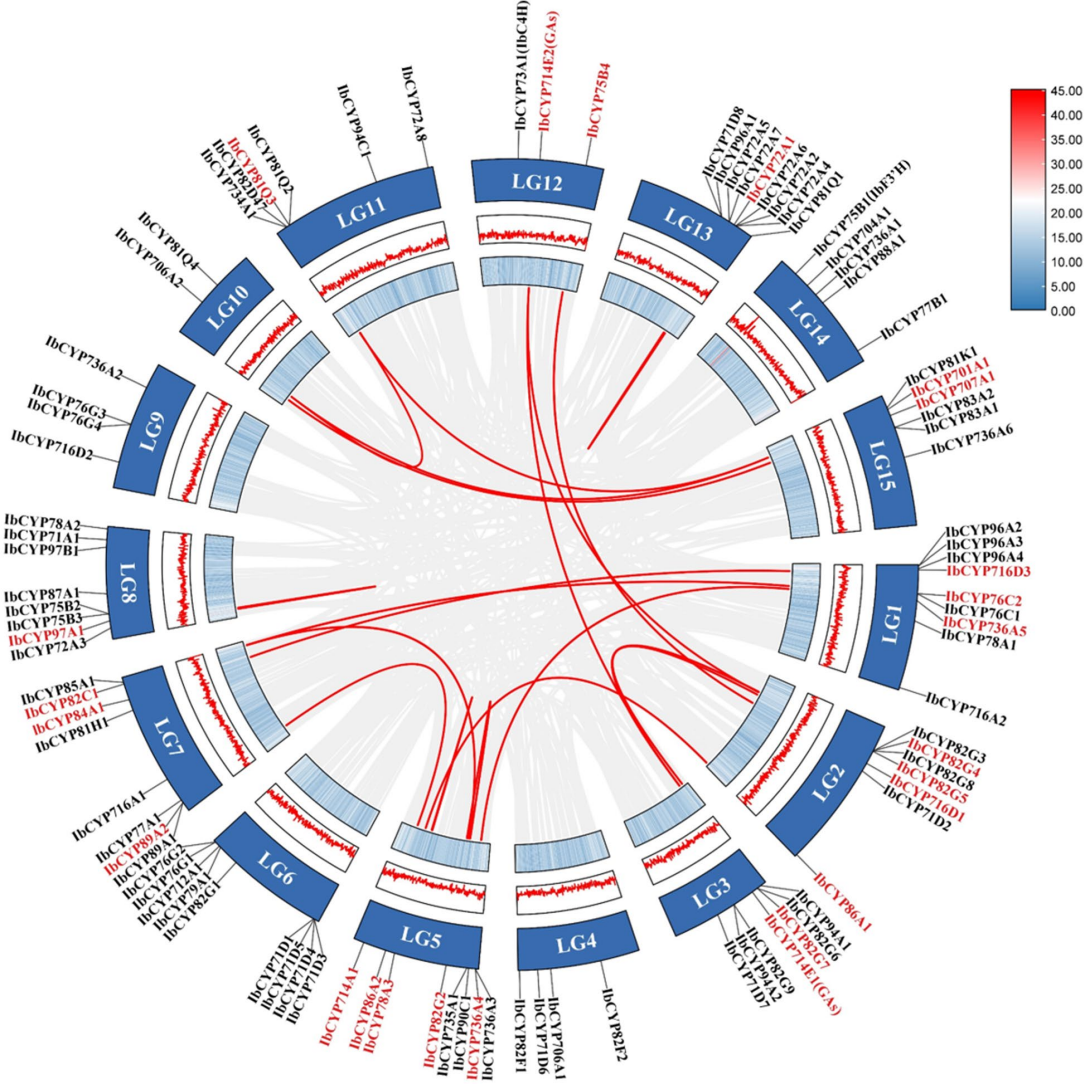
Distribution and collinearity of the *IbCYP* gene family in the sweet potato genome. *IbCYPs* labeled with red had collinearity, while those labeled with black had no collinearity. The two rings in the middle represent the gene density of each chromosome. The gray background lines represent a collinear background and the red lines indicate a collinear relationship between *IbCYP* members

After analyzing the chromosomal localization, we found that the 95 *IbCYP* genes were spread across 15 chromosomes (Fig. 4). Chromosomes 1 (LG1), 6 (LG6), and 13 (LG13) had the highest number of genes, with nine *IbCYP* genes each. Chromosomes 5 (LG5), 7 (LG7), and 8 (LG8) followed closely with eight *IbCYP* genes each. Chromosomes 2 (LG2) and 3 (LG3) contained seven *IbCYP* genes. In contrast, chromosomes 10 (LG10) and 12 (LG12) had the fewest genes, with only two and three genes, respectively. Additionally, we identified 15 pairs of tandemly duplicated genes among the *IbCYP* genes. These genes were located close to each other on the chromosomes and formed clusters on the phylogenetic tree, indicating similar functions. The expansion of the gene family was mainly attributed to tandem duplication and segment duplication, as shown by the presence of 13 duplicate gene pairs distributed on different chromosomes through MCScanX collinearity analysis (Fig. 5). This suggests that tandem duplication and segment duplication played a role in the expansion of *CYP450* genes.

### Synteny analysis of *IbCYP* genes in sweet potato, pepper, tomato, rice, maize, and *Arabidopsis*

**Fig. 6:**
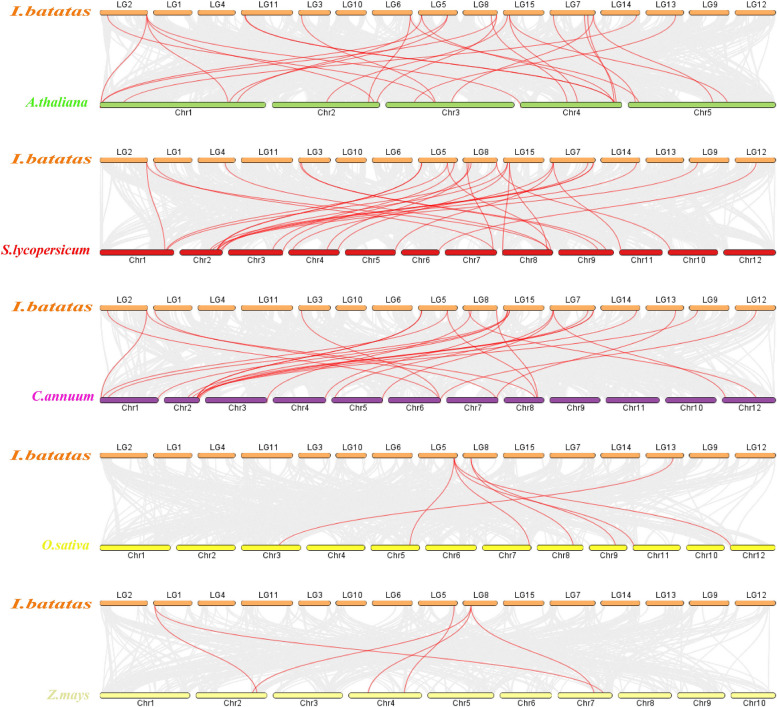
Collinearity analysis of CYP450 proteins in sweet potato among species. The species were Arabidopsis, tomato, pepper, maize, and rice. The red line represents the homologous *CYP450 *gene pair of the plant genome, and the gray line represents the collinear block of the plant genome

 To better understand how the *CYP450* family evolved in sweet potatoes compared to other species, we conducted an evolutionary relationship analysis of *CYP450* genes. Specifically, we compared sweet potatoes with three dicotyledonous plants (*Arabidopsis*, tomato, and pepper) and two monocotyledonous plants (maize and rice) (Fig. 6). The analysis revealed that sweet potatoes shared 27 collinear genes with Arabidopsis, and 31, 26, 7, and 6 collinear genes with tomato, pepper, rice, and maize, respectively. These findings suggest that the *IbCYPs* in sweet potatoes have a close evolutionary relationship with the *CYP450* genes in dicotyledonous plants, particularly with tomatoes and peppers from the Solanaceae family.

### Analysis of cis‑regulatory element distribution in *IbCYP* promoters

**Fig. 7:**
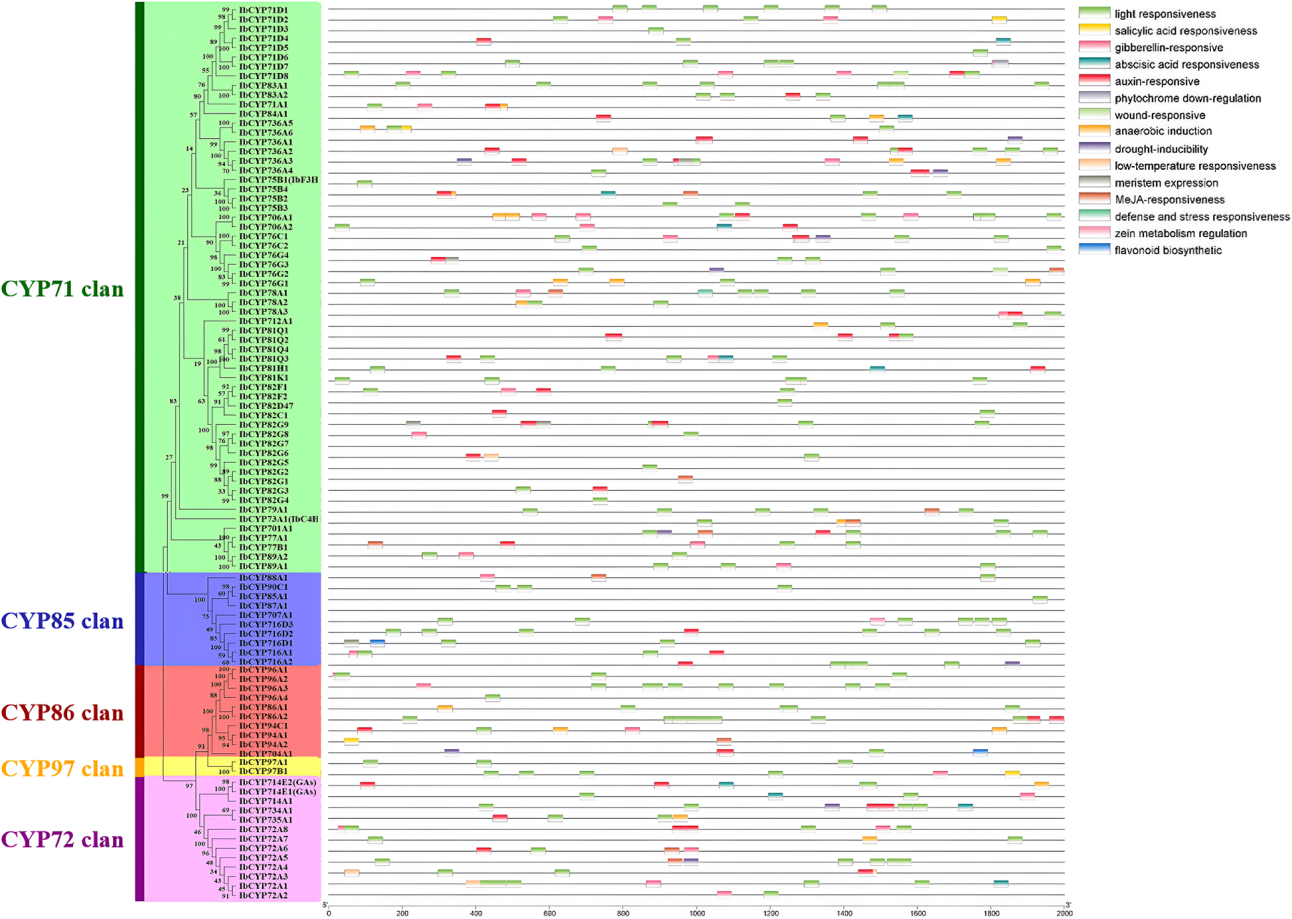
Distribution of cis-acting elements of the *IbCYP* gene family in sweet potato. Distribution of cis-acting elements identified in the 2000 bp upstream promoter region of the sweet potato *IbCYP* gene

We extracted the genomic sequence of the *IbCYP* genes upstream region, specifically 2000 base pairs, to study their potential biological functions. This sequence was considered a hypothetical promoter sequence for cis-acting element analysis (Fig. 7). Our analysis revealed the presence of different types of cis-acting elements in the *IbCYP* gene family, which are associated with plant growth and development, hormone responses, and responses to abiotic stress. When examining the genes, it was found that 91 genes contained one to ten light response elements, 39 genes contained one to four auxin response elements, and 30 genes contained one to three gibberellin response elements.

Moreover, some genes contained cis-acting elements related to plant hormones and abiotic stress, such as MeJA-responsive elements, salicylic acid response elements, abscisic acid response elements, and elements responding to drought, hypoxia, and low temperature. Additionally, a few genes had cis-acting elements linked to plant secondary metabolism and growth development, involving zein metabolism regulation, flavonoid biosynthesis, endosperm and meristem expression, and phytochrome downregulation response elements. Two genes also contained wound-responsive elements. Overall, the promoter regions of these *IbCYPs* contained various types of cis-elements, indicating their potential involvement in diverse biological processes and regulatory pathways.

### Transcript factors networks of *IbCYP* genes

**Fig. 8:**
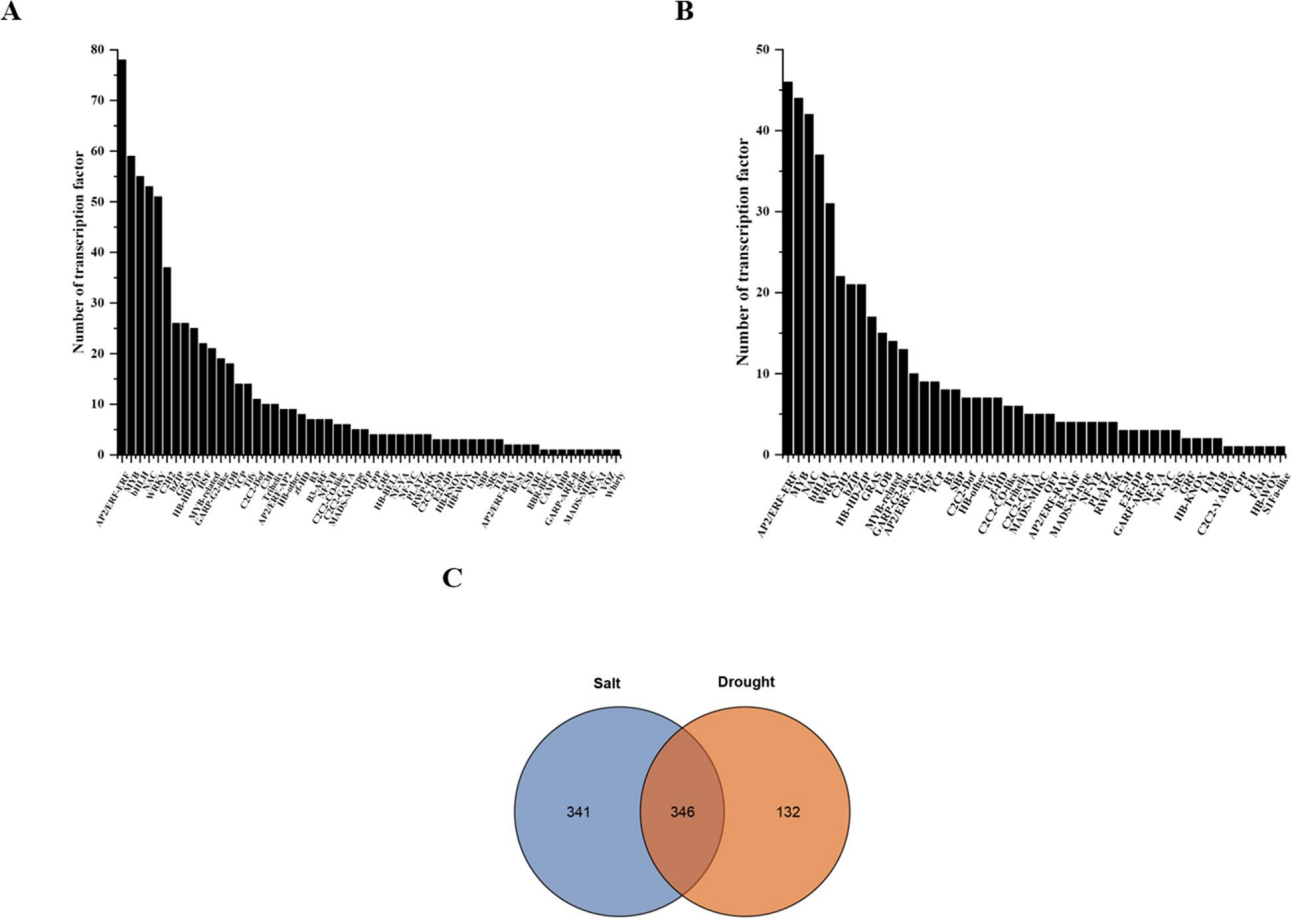
The putative transcription factor regulatory network analysis of *IbCYP* genes. **A** Distribution of putative transcription factor regulatory networks of the *IbCYP* gene under salt stress. **B** Distribution of putative transcription factor regulatory networks of the *IbCYP* gene under drought stress. **C** Statistical analysis of the differences in putative transcription factor regulatory networks of the *IbCYP* gene under salt and drought stress

Through analysis of potential transcription factors (TFs), it was found that a total of 687 TFs were identified in the *IbCYP* genes under salt stress, distributed among 56 different TF families, such as AP2/ERF-ERF, MYB, bHLH, NAC, WRKY, C2H2, bZIP, GRAS, and others (Fig. 8A). In the *IbCYP* genes under drought stress, a total of 478 TFs were identified, distributed among 48 different TF families, including AP2/ERF-ERF, MYB, NAC, bHLH, WRKY, C2H2, bZIP, HB-HD-ZIP, GRAS, LOB, and others (Fig. 8B). The analysis of TF quantity revealed that there are 346 common TFs shared between salt stress and drought stress (Fig. 8C).

### Regulatory network in sweet potato

**Fig. 9:**
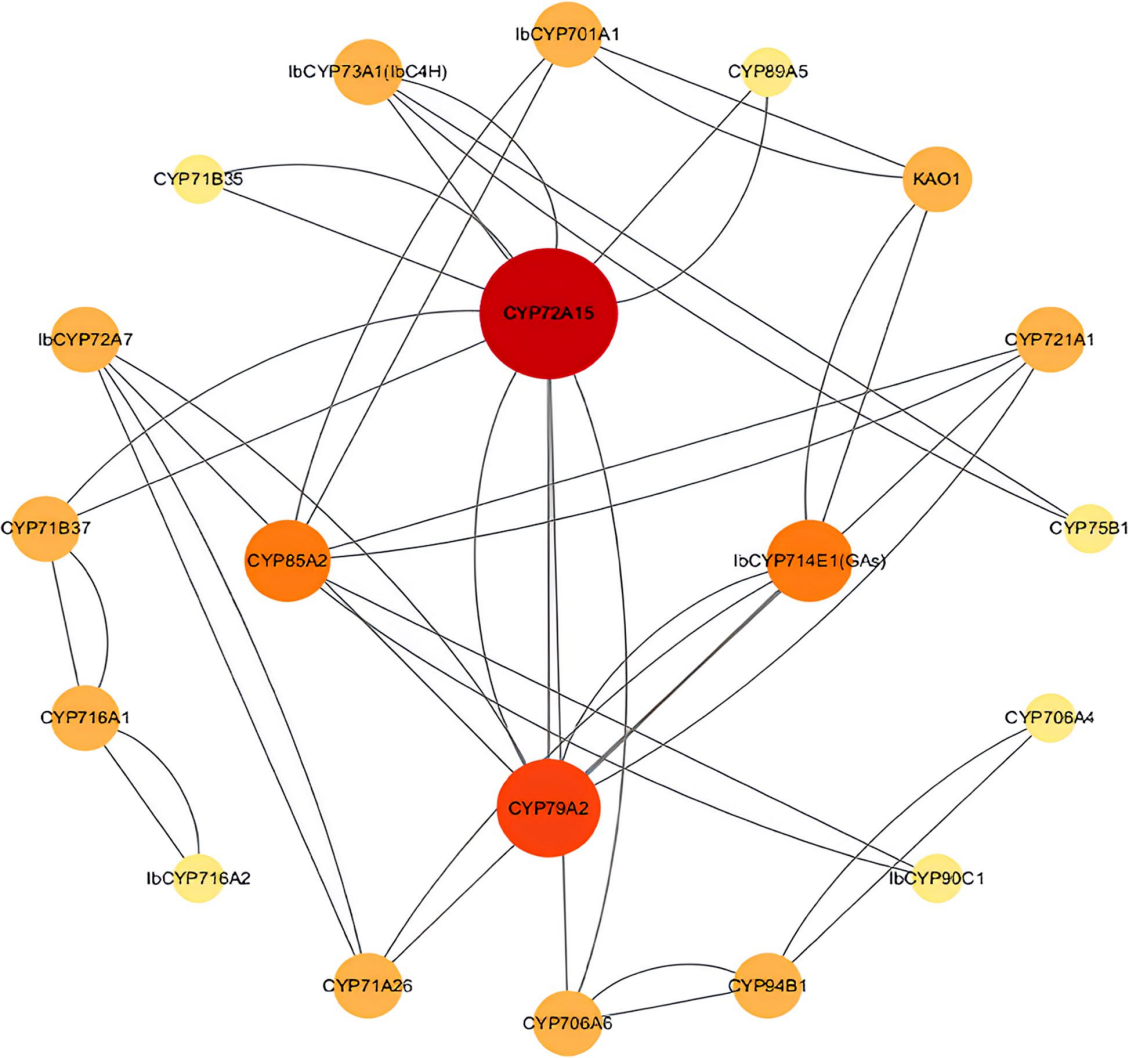
rotein–protein interaction (PPI) network of significant genes in sweet potato. Nodes represent proteins, central nodes are indicated in red, and black lines indicate interactions between nodes. The darker the color, the more important the protein in the interaction network

We used the STRING database to predict potential interactions among the IbCYP proteins (Fig. 9). There were 20 nodes in the IbCYP protein interaction network, each of which interacted with other nodes. Some proteins exhibited direct interactions, such as *IbCYP706A2* and *IbCYP94A2*, whereas others exhibited more complex multigene interactions, such as *IbCYP714E1 (Gas)* and *Ib*CYP79A1. Notably, *IbCYP72A8* was predicted to be central nodes, radiating six connections to other genes.

### Expression patterns of *IbCYP* genes in sweet potato

**Fig. 10:**
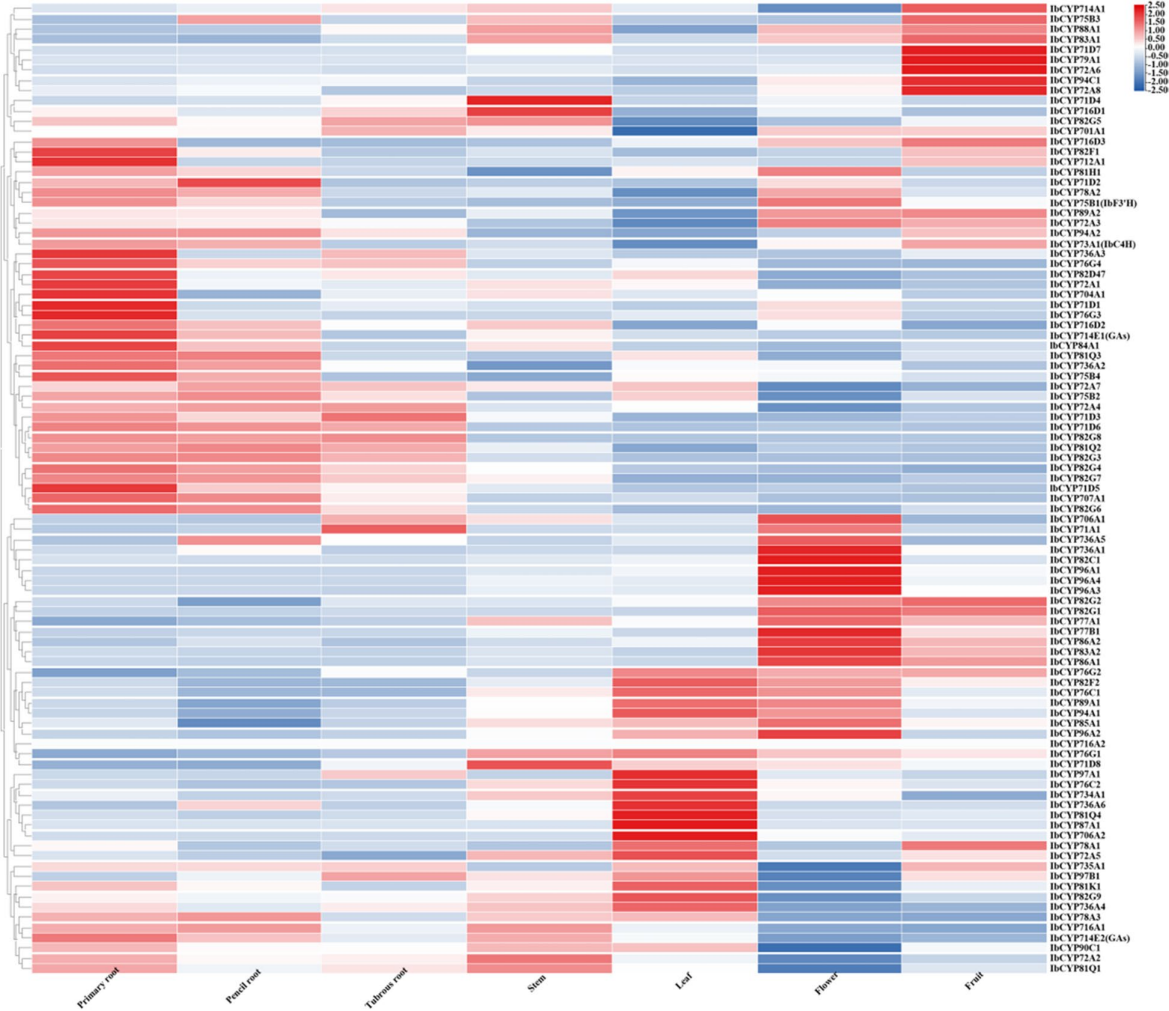
Expression heatmap of the *IbCYP* gene in different tissues of sweet potato. Red and blue indicate the intensity of genes in the heatmap: the more intense the red color is, the higher the gene expression level, while the more intense the blue color is, the lower the gene expression level

We analyzed the expression patterns of the *IbCYP* genes in various plant tissues using transcriptome data. The expression levels were measured as fragments per kilobase of exon model per million mapped fragments (FPKM). Our findings indicate that 95 *IbCYP* genes showed significant differences in expression patterns across different tissues (Fig. 10). Specifically, three genes exhibited high expression levels in tuber roots, 13 genes in leaves, 14 genes in flowers, and 9 genes in fruits.

**Fig. 11:**
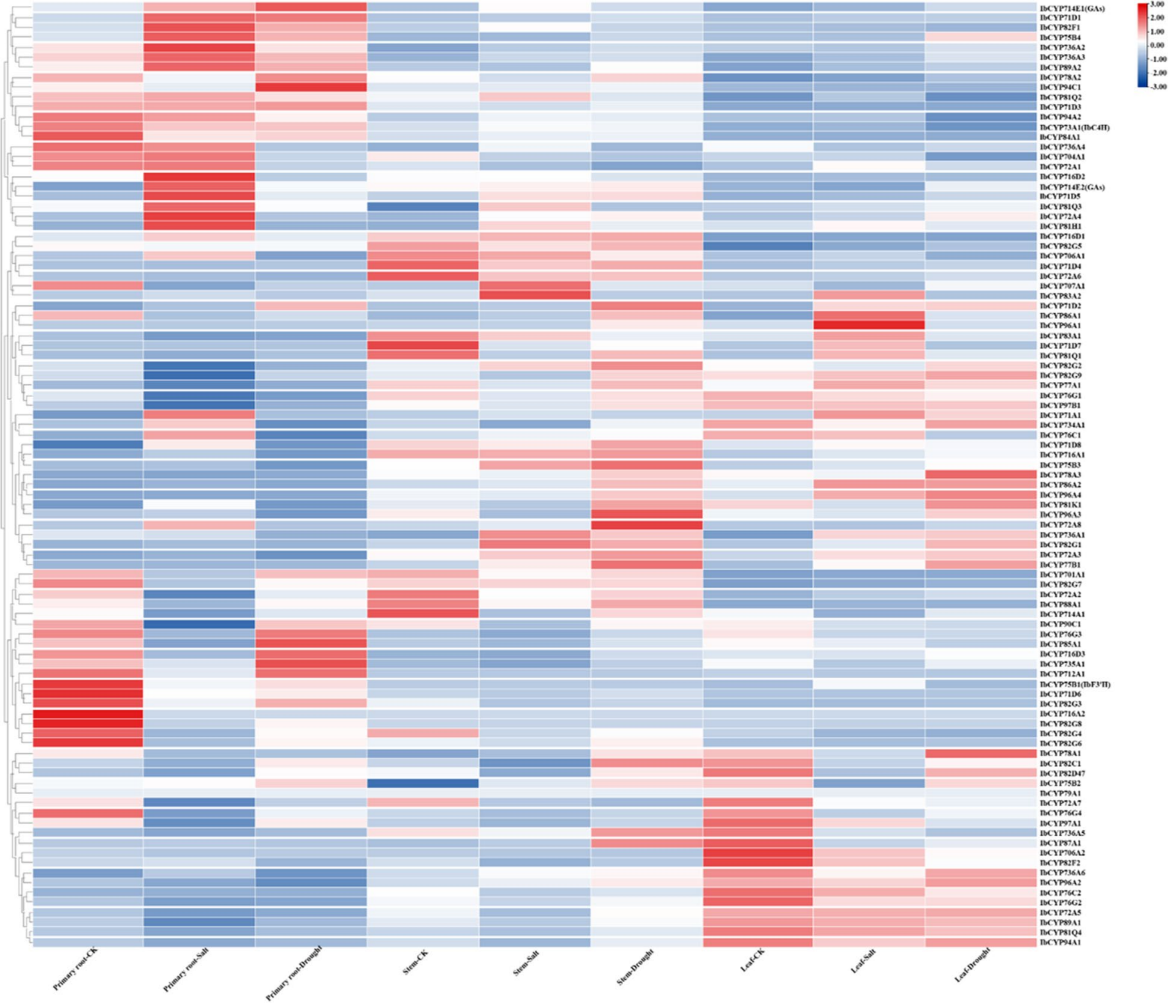
Expression heatmap of *IbCYP* in root, stem, and leaf tissues of sweet potato under salt and drought stress. Red and blue indicate the intensity of genes in the heatmap: the more intense the red color is, the higher the gene expression level, while the more intense the blue color is, the lower the gene expression level

We also examined the expression of *IbCYP* genes under salt and drought stress conditions in sweet potato tissues (Fig. 11). In tissues exposed to salt and drought stress, the expression levels of all 95 *IbCYP* genes showed significant differences. Specifically, under salt stress, 21 genes were upregulated and 28 genes were downregulated in root tissues. In stem tissues, 15 genes were upregulated and 11 genes were downregulated, while in leaf tissues, 5 genes were upregulated and 10 genes were downregulated. Under drought stress, 9 genes were upregulated 22 genes were downregulated in root tissues, 20 genes were upregulated and 4 genes were downregulated in stem tissues, 13 genes were upregulated and 11 genes were downregulated in leaf tissues (Table 2). These findings suggest that *IbCYP* genes have distinct expression patterns under salt and drought stress conditions. Overall, most of the genes responded to different stress conditions.

**Table 2:** The regulation of the *IbCYP* gene under salt and drought stresses

	down	up		down	up
Under salt stress			Under drought stress		
Root	28	21	Root	22	9
Stem	11	15	Stem	4	20
Leaf	10	5	Leaf	11	13

**Fig. 12:**
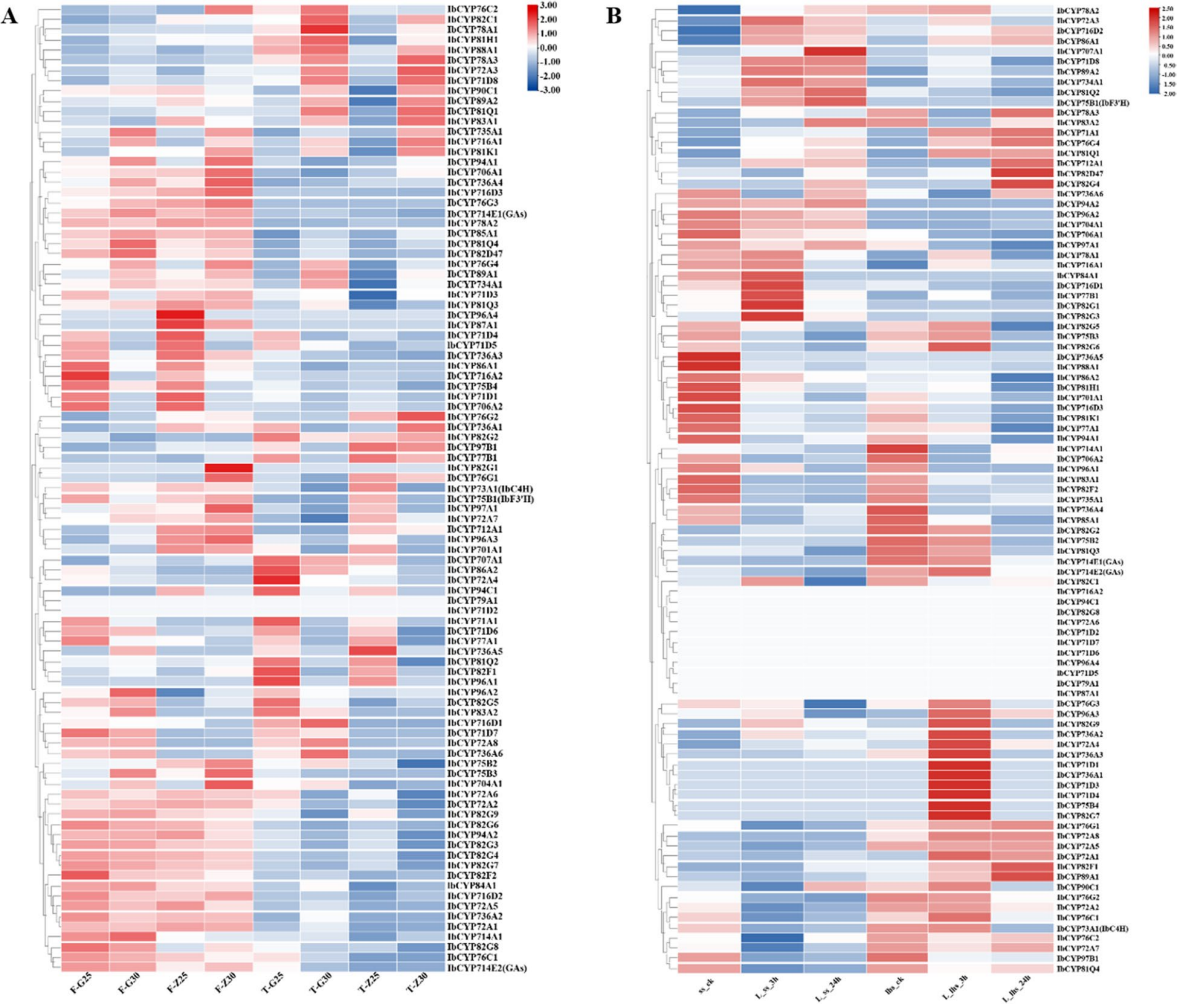
**A** Gene expression patterns of *IbCYP* genes under heat stress as determined by RNA-seq. F: fibrous roots; T: tuberous roots; Z: heat-sensitive “Ziluolan”; G: heat-tolerant “Guangshu 87”. **B** Gene expression patterns of *IbCYP* genes under cold stress as determined by RNA-seq. ss: clod-sensitive “Shenshu 28”, lhs: cold-tolerant “Liaohanshu 21”

The expression of 95 *IbCYP* genes was detected under high-temperature stress, and we focused on two specifically highly expressed genes, including *IbCYP82G1* in “Ziluolan” fibrous roots and *IbCYP78A1* in “Guangshu 87” roots (Fig. 12A). These two genes may be related to the heat tolerance of sweet potatoes. Similarly, the expression of 95 *IbCYP* genes was detected under cold stress (Fig. 12B). In “Shenshu 28”, after 3 h of cold stress, *IbCYP82G1* and *IbCYP82G3* were highly expressed, but after 24 h of cold stress, the expression levels of these two genes decreased, while *IbCYP707A1* showed specific high expression. In “Liaohanshu 21”, after 3 h of cold stress, seven genes including *IbCYP82G7* were highly expressed, but after 24 h of cold stress, the expression levels of these seven genes decreased, while *IbCYP82D47* and *IbCYP82G4* were highly expressed.

**Fig. 13:**
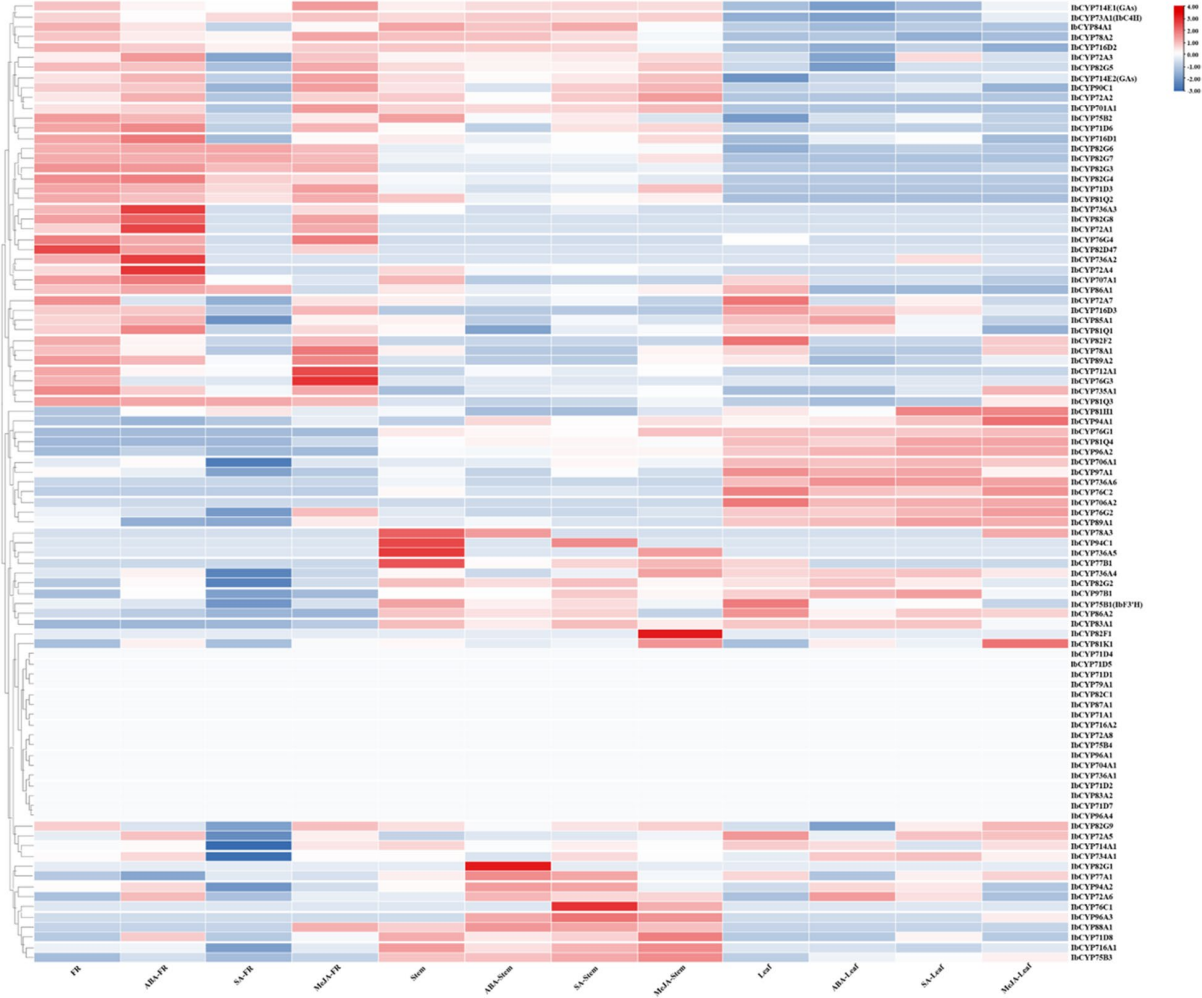
Expression analysis of *IbCYP* genes in fibrous roots (FR), stems and leaves of sweet potato under hormones treatment as determined by RNA-seq

The expression profiles of 95 *IbCYP* genes were identified in three distinct tissues using the “Xushu 18” RNA-seq data obtained from the NCBI database (PRJNA511028) (Fig. 13). In fibrous roots, *IbCYP736A3*, *IbCYP736A2*, and *IbCYP72A4* were highly expressed after ABA treatment, while *IbCYP76G3* and *IbCYP712A1* were highly expressed after MeJA treatment. In stems, *IbCYP82G1* showed specific high expression after ABA treatment, *IbCYP76C1* was highly expressed after SA treatment, and *IbCYP82F1* showed specific high expression after MeJA treatment.

### Quantitative analysis of *IbCYP* genes in different tissues

**Fig. 14:**
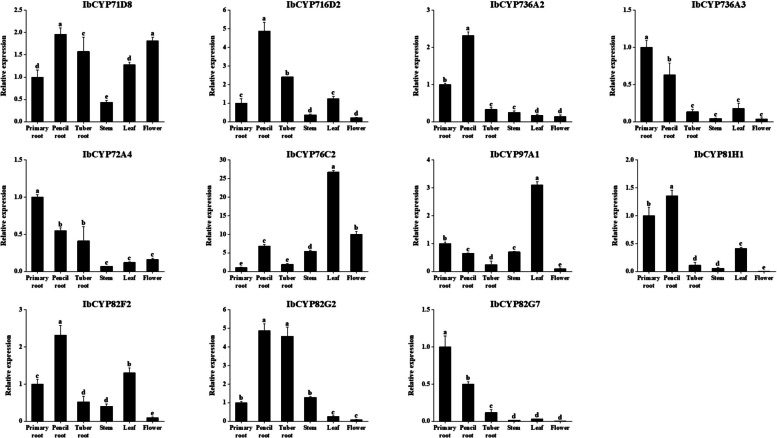
Expression patterns of 11 *IbCYP* genes in different tissues. The x-axes represent different tissues including primary root, pencil root, tuber root, stem, leaf, and flower; the y-axes indicate the relative expression of *IbCYP* genes. The different letters of a, b, c, d, and e indicate significant differences at p < 0.05, as determined by one-way ANOVA with SPSS single-factor tests

To confirm the accuracy of the transcriptome data, we selected 11 genes that showed significant expression differences and performed qRT-PCR analysis (Fig. 14). The results of the expression analysis of *IbCYP* genes in different parts of sweet potato were consistent with the transcriptome data. In general, the expression of these *IbCYP* genes was primarily detected in the pencil roots and leaves of sweet potatoes. Additionally, there were noticeable differences in the expression of these *IbCYP* genes among different parts. Notably, *IbCYP82G2* exhibited the highest expression level in the tuber, while *IbCYP82G7* showed the highest expression level in the primary root. This suggests that genes belonging to the same subfamily in the *CYP450* family may have diverse functions.

### Quantitative analysis of *IbCYP* genes under abiotic stresses

**Fig. 15:**
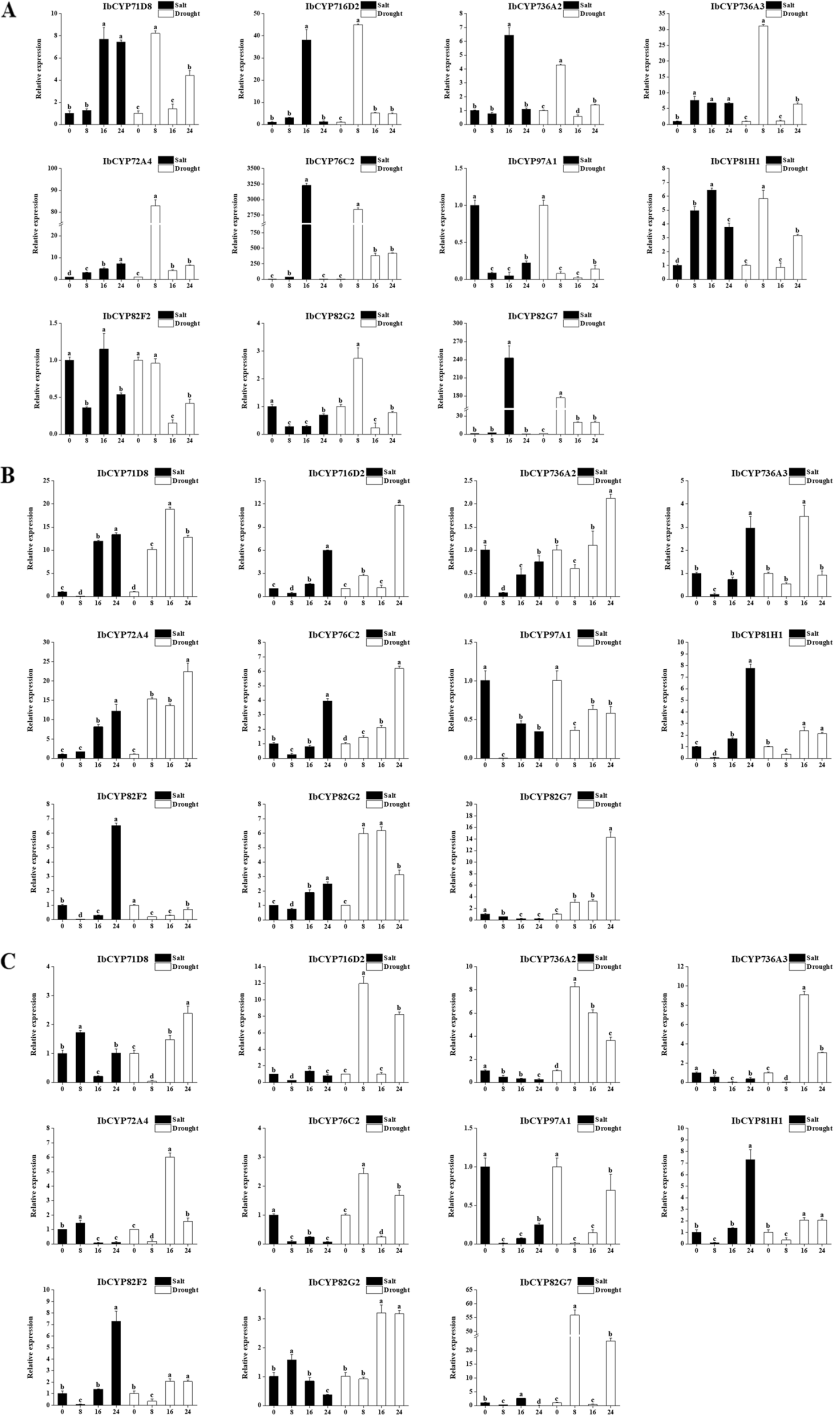
Changes in the expression levels of 11 IbCYP genes in different tissues under salt and drought treatments. The different letters of a, b, c, and d indicate significant differences at p < 0.05, as determined by one-way ANOVA with SPSS single-factor tests. **A** Expression of 11 genes in primary roots under salt and drought stress. **B** Expression of 11 genes in stems under salt and drought stress. **C** Expression of 11 genes in leaves under salt and drought stress

To evaluate the expression of *IbCYP* genes in different tissues of sweet potato under various stress conditions, we utilized a technique called quantitative real-time polymerase chain reaction (qRT-PCR). The expression levels of sweet potato were examined after subjecting them to different durations of stress (Fig. 15). The results showed that exposure to salt and drought stress caused an increase in *IbCYP* gene expression in different parts of sweet potato. Specifically, under both stress conditions, most *IbCYP* genes in the primary root initially showed an increase in expression followed by a decrease, indicating a consistent pattern of expression. However, the expression of these *IbCYP* genes in stems showed the opposite trend. In the case of sweet potato leaves, a more complex pattern of expression was observed. For instance, *IbCYP736A2* displayed a gradual decrease in expression under salt stress but showed an initial increase followed by a decrease under drought stress. Additionally, it is important to note that the highest expression level of *IbCYP76C2* in roots was 3230 times higher than that in the control group after 16 h of salt stress, and 2844 times higher after 8 h of drought stress. Similarly, the highest expression level of *IbCYP82G7* in roots was 242 times higher than that in the control group after 16 h of salt stress and 177 times higher after 8 h of drought stress.

## Discussion

Plant *CYP450* genes are involved in the catalysis of multiple reactions, including growth, development, and secondary metabolite biosynthesis pathways [64–67]. With the development of gene sequencing technology, an increasing number of plant genomes have been deciphered, and the identification of the *CYP450* family is expanding to more plants, such as tea tree, foxtail millet, citrus, and peanut, etc. [68–72]. Due to gene duplication and divergence events, higher plants possess a large number of *CYP450* genes. In this study, we employed more rigorous criteria to identify *CYP450* genes in sweet potatoes. Our methodology included local BLAST, HMMER, CDD, and conserved motif analysis. To enhance accuracy, we refined the coding sequence (CDS) and amino acid sequences of 95 *IbCYP* genes using existing transcriptome sequencing data from our laboratory. In terms of gene structure, sweet potato genes show a wide range of gene lengths and significant differences in the number of exons. This may be related to the abundant biological functions carried out by the *CYP450* superfamily. The sweet potato genome encompasses a total of 95 *CYP450* genes, providing a valuable genetic foundation for further investigations into their functions.

In sweet potatoes, the predicted 95 *CYP450* genes are classified into 5 gene families, namely, the *CYP71* family, *CYP72* family, *CYP85* family, *CYP86* family, and *CYP97* family, totaling 31 subfamilies. Among them, 60 (63.1%) genes belonging to the *CYP71* clan were classified as type A, while the rest were classified as nontype A. In contrast, more members of the *CYP450* gene family have been identified in other crops. For example, 326 *CYP450* family members were identified in rice, 332 *CYP450* genes in soybean, and 478 *CYP450* genes in pepper [7, 42, 43]. This could be attributed to the fact that sweet potato’s two genomes are too similar. In the future, there may be additional discoveries of *CYP450* family members in sweet potatoes. Conducting a collinearity analysis within a specific species provides insights into the homology of genes across different chromosomes. Through chromosome localization and collinearity analysis, it was confirmed that *IbCYP*, a specific gene, consists of 28 duplicated gene pairs. Among these pairs, 15 were classified as tandem duplications, while the remaining 13 were classified as segmental duplications. Thus, it is speculated that the expansion of the *CYP450* gene family in the evolutionary process was primarily driven by segmental duplications, with tandem duplications playing a supplementary role. In addition, a collinearity analysis was performed among different species to explore gene evolution and genetic relationships. The evolutionary relationship between sweet potato and other species was examined based on family genes using a collinear analysis. The results revealed that the *CYP450* family genes of sweet potato were more closely related to other Solanales plants, such as tomato and pepper. Specifically, 31 and 26 collinear gene pairs were identified in tomato and pepper, respectively. However, the genetic relationship between sweet potato and gramineous plants, such as maize and rice, was found to be less significant, as only a few collinear gene pairs were observed. These findings align with the results obtained from the genetic relationship analysis.

The cis-acting elements of promoters play a vital role in the regulation of gene expression. In this study, we verified that the *IbCYP* promoter region of sweet potato contained several elements related to the hormone regulation pathway. Among them, light responsiveness, auxin responsiveness, and gibberellin responsiveness were detected in most genes. Therefore, we inferred that light, gibberellin, and auxin may influence *IbCYP* gene expression, thereby affecting the growth and development of sweet potatoes. In *Arabidopsis*, *CYP714A1* and *CYP714A2* may function in the early stages of the GA biosynthetic pathway [73]. These drought-inducing elements were detected in 10 sweet potato genes. The results from the heatmap analysis revealed that *IbCYP701A1* and *IbCYP96A3* were highly expressed under drought stress. These findings indicated that *IbCYP* gene expression was regulated by cis-elements related to plant development and abiotic stress tolerance.

The expression pattern of genes reflects their functions to a certain extent. Therefore, in this study, we analyzed the expression patterns of the *IbCYP* genes. In sweet potatoes, the *CYP71* clan exhibits specific expression in multiple tissues. For example, *IbCYP71D5* is specifically expressed in primary roots; *IbCYP71D8* is specifically expressed in stems; and *CYP76C2* in Arabidopsis has been found to respond to leaf senescence and aging in cell cultures, which are related to cellular deterioration and eventual cell death [74]. Similarly, *IbCYP76C2* was detected to exhibit leaf-specific expression in sweet potato. It is speculated that it may also play a similar role in sweet potato leaves; *IbCYP77B1* is specifically expressed in the flowers, and *IbCYP71D7* is specifically expressed in the fruits. The monogenic family *CYP97* clan consists of two genes, namely *IbCYP97A1* and *IbCYP97B1*. This is similar to the case of pepper (3 genes) [42]. These two genes are specifically expressed in the leaves and stems of sweet potato, indicating a potential relationship with the growth and development of sweet potato stems and leaves. *IbCYP77A1* shows high expression in both flowers and fruits of sweet potatoes, which is similar to the role of *CYP77A4* in *Arabidopsis thaliana* [75], where it is involved in the development of cotyledons. This suggests that *IbCYP77A1* may play a role in the reproductive development of sweet potatoes. Under environmental stress, *IbCYP* genes also play a regulatory role. Usually, the root system is the first part to be affected by environmental stress. As the heatmap shows, the *IbCYP* genes were generally highly expressed in the primary root under different stresses. In sweet potatoes, a total of 34 *IbCYP* genes are upregulated in response to salt and drought stress. In response to cold and heat stress, *IbCYP450* genes also show a response. *IbCYP82G1*, *IbCYP82G3*, and *IbCYP707A1* exhibit different levels of response under cold stress. Under heat stress, both *IbCYP82G1* and *IbCYP78A1* are highly expressed, but the expression patterns of these two genes may vary among different varieties, possibly due to intervarietal differences. The *IbCYP450* genes also show varying degrees of response to plant hormones, which corresponds to the presence of multiple plant hormone response elements in the promoter cis-acting elements of *IbCYP450* genes. These findings have also been corroborated in foxtail millet. However, the response of *CYP450* genes to low temperature, salt stress, and plant hormones differs between foxtail millet and sweet potato, which is likely due to genetic differences between the two species [68]. It has been reported that the *CYP86* clan has a positive regulatory effect on the plant immune system [76]. *CYP86* clan genes were expressed in the roots and leaves and were related to drought and salt tolerance.

## Conclusion

We identified 95 *IbCYP* genes in the sweet potato genome, which were classified into 5 families and 31 subfamilies. Our evolutionary analysis of the *CYP450* superfamily in sweet potato showed that the *IbCYP* genes have undergone frequent duplication and functional diversification. This will help us understand the complex agronomic traits and evolutionary processes of sweet potatoes. Additionally, we observed species- or family-specific expansions of the *CYP450* superfamily, which may explain species divergence events. Expression analysis revealed the diversified expression patterns of *CYP450* genes in sweet potatoes, which were expressed in different tissues, under various abiotic stress conditions, and in response to plant hormones. This indicates the functional diversity and regulation of *CYP450* genes in sweet potatoes. The results of this study provide a solid foundation for further exploring the molecular evolution mechanisms and potential functions of the *CYP450* gene family in sweet potatoes.

### Supplementary Information

**Additional file 1.**

## Data Availability

All datasets supporting the results of this study are included in this article and its Supplementary data files. The transcriptomic data used in this study can be accessed through the NCBI accession numbers PRJNA511028, PRJNA987163, and PRJNA744414. The Ipomoea Genome Hub website (https://ipomoea-genome.org/). The Cytochrome P450 Homepage website (http://drnelson.uthsc.edu/CytochromeP450.html). The Pfam database (http://pfam.sanger.ac.uk). The ExPASy ProtParam tool (http://web.ExPASy.org/protparam/). The BUSCA (http://busca.biocomp.unibo.it/). The online website GSDS2.0 (http://gsds.gao-lab.org/). The MEME online website (http://meme-suite.org/tools/meme). The PlantCARE website (https://bioinformatics.psb.ugent.be/webtools/plantcare/html/). The online STRING database (https://string-db.org/).
